# Automated and partly automated contact tracing: a systematic review to inform the control of COVID-19

**DOI:** 10.1016/S2589-7500(20)30184-9

**Published:** 2020-08-19

**Authors:** Isobel Braithwaite, Thomas Callender, Miriam Bullock, Robert W Aldridge

**Affiliations:** a UCL Public Health Data Science Research Group, Institute of Health Informatics, University College London, London, UK; b Department of Applied Health Research, University College London, London, UK; c UCL Collaborative Centre for Inclusion Health, University College London, London, UK

## Abstract

Evidence for the use of automated or partly automated contact-tracing tools to contain severe acute respiratory syndrome coronavirus 2 is scarce. We did a systematic review of automated or partly automated contact tracing. We searched PubMed, EMBASE, OVID Global Health, EBSCO Medical COVID Information Portal, Cochrane Library, medRxiv, bioRxiv, arXiv, and Google Advanced for articles relevant to COVID-19, severe acute respiratory syndrome, Middle East respiratory syndrome, influenza, or Ebola virus, published from Jan 1, 2000, to April 14, 2020. We also included studies identified through professional networks up to April 30, 2020. We reviewed all full-text manuscripts. Primary outcomes were the number or proportion of contacts (or subsequent cases) identified. Secondary outcomes were indicators of outbreak control, uptake, resource use, cost-effectiveness, and lessons learnt. This study is registered with PROSPERO (CRD42020179822). Of the 4036 studies identified, 110 full-text studies were reviewed and 15 studies were included in the final analysis and quality assessment. No empirical evidence of the effectiveness of automated contact tracing (regarding contacts identified or transmission reduction) was identified. Four of seven included modelling studies that suggested that controlling COVID-19 requires a high population uptake of automated contact-tracing apps (estimates from 56% to 95%), typically alongside other control measures. Studies of partly automated contact tracing generally reported more complete contact identification and follow-up compared with manual systems. Automated contact tracing could potentially reduce transmission with sufficient population uptake. However, concerns regarding privacy and equity should be considered. Well designed prospective studies are needed given gaps in evidence of effectiveness, and to investigate the integration and relative effects of manual and automated systems. Large-scale manual contact tracing is therefore still key in most contexts.

## Introduction

In response to the rapid spread of severe acute respiratory syndrome coronavirus 2 (SARS-CoV-2) since December, 2019, governments worldwide have applied widespread physical distancing measures to attempt to curb transmission.^1^ These policies have suppressed case numbers^2^, ^3^ but have substantial economic, social, and indirect health consequences,^4^ leading to a growing focus on alternative control strategies.^5^

Contact tracing is a well established part of the management of infectious disease outbreaks, which aims to interrupt chains of infection transmission (eg, through quarantining contacts), and has formed part of the response to the COVID-19 pandemic in many countries.^6^, ^7^ Traditionally, contact tracing involves a person recalling their recent close contacts and activities. Individuals who are deemed to be at risk of infection (on the basis of contact definitions that might vary by country and change over time) are then contacted and advised to take action to reduce onward transmission—eg, to self-quarantine for a specified time period.^8^ The ability of any contact-tracing system to reduce disease transmission depends on timely detection and isolation of index cases (which requires rapid, population-level, active surveillance);^9^ how quickly and comprehensively the system can identify and (if applicable) advise quarantine of contacts who will go on to become infected, relative to the infectious period of the disease in question; and quarantine adherence.^10^, ^11^, ^12^ A UK report estimated that manual contact tracing of non-household members would reduce the number of new infections occurring by 5–15%, in addition to the effect of quarantining symptomatic individuals and their household members.^13^

Typically, the limitations of contact tracing include incomplete or incorrect recall of contact events by cases; the time taken to notify contacts manually, which can delay quarantine;^14^ and the fact that it is often resource intensive and time consuming.^12^, ^15^ Technology could be used to address some of these limitations, including by automating the processing of test results or symptom reports and by use of smartphone capabilities (eg, Bluetooth) to identify and notify contacts instantaneously who are at risk of infection.^14^, ^16^, ^17^ Automated contact tracing for COVID-19 has been deployed in several countries,^18^, ^19^ but in the UK, its introduction has been delayed by technical setbacks.^20^ The practical, technical, legal, and ethical considerations involved are complex;^20^, ^21^, ^22^ and uptake, privacy, security, and testing access have been identified as potential barriers to effectiveness.^21^, ^23^

This systematic review aims to assess the effectiveness of automated and partly automated contact-tracing systems (those that involve some automation within contact-tracing processes, but that do not use a device to gather data as a proxy for contact or do require users to notify contacts themselves) in identifying contacts who are at risk and in controlling disease transmission in humans. These factors should inform discussions about the balance between the benefits and potential risks of automated contact tracing in controlling COVID-19.

## Methods

### Search strategy and selection criteria

We searched PubMed, EMBASE, and OVID Global Health for articles from any setting published between Jan 1, 2000, and April 14, 2020. We supplemented these findings with searches of medRxiv, bioRxiv, arXiv, EBSCO Medical COVID Information portal, Cochrane Library, and Google Advanced (appendix pp 1–4) and scanned relevant references of included studies. We also included studies identified through professional networks up to April 30, 2020. This systematic review is registered with PROSPERO (CRD42020179822) and was done in line with the Preferred Reporting Items for Systematic Reviews and Meta-Analyses reporting standards.^24^ The protocol is available as a preprint.^25^

Primary outcomes of interest were the number or proportion of contacts identified and the number or proportion of contacts who go on to become infected that are identified (where the term contacts refers to people considered to be at risk because of their exposure to a person who was infected). Secondary outcomes included the effect on either the basic reproduction number (R_0_; the average number of secondary cases infected by one infectious person in a completely susceptible population) or the effective reproduction number (R_e_; the average number of secondary cases infected by one infectious person in a real world population), or other indicators of outbreak control (eg, completeness or timeliness of contact follow-up or intervention). Additionally, secondary outcomes included population uptake (ie, app uptake or participation); resource requirements (eg, time, financial resources, testing capacity, training, or specific expertise) or cost-effectiveness (eg, cost per case prevented or per quality-adjusted life-year); and ethical considerations and lessons learnt from implementation of an automated or partly automated contact-tracing system. Our original protocol included data security, privacy issues, and public perception, but was modified to exclude these outcomes, partly because they are addressed by the Ada Lovelace Institute report,^21^ which was published during our review process, and partly to focus on the evidence of effectiveness from a public health perspective.

We included interventional, observational, modelling, and case studies related to automated or partly automated contact tracing in humans that reported findings regarding at least one outcome of interest. We included studies of COVID-19, severe acute respiratory syndrome, Middle East respiratory syndrome, influenza, or Ebola virus, or, in modelling studies, hypothetical infections spread through respiratory transmission. Studies in which some contact-tracing processes were automated (eg, automated calculation or updating of follow-up periods, contact list generation, alert generation, or transmission mapping), but did not use data from a device as a proxy for contact or that did require users to notify contacts, were considered partly automated. Study designs that were purely qualitative were excluded, as were app protocols and studies of monitoring during quarantine. Articles with or without comparators were considered eligible. Both peer-reviewed articles and preprint and grey literature articles that were not peer-reviewed were included.

Our search was restricted to manuscripts in English, and studies available only as an abstract (eg, conference abstracts) were excluded. Non-English language studies flagged for full text review have been collated in the appendix (appendix p 17). Titles and abstracts were screened by a reviewer (IB or TC), with 10% of excluded records dual screened. Full texts were screened for eligibility by two reviewers (IB and MB or TC). Discrepancies were resolved by consensus, with an independent view given by a third reviewer (TC or RWA). All exclusion decisions were documented.

### Data analysis

One reviewer (IB) extracted data (details in the protocol^25^) using a standardised spreadsheet that had been pilot tested. Data extraction was reviewed for each study by a second reviewer (MB or TC). One reviewer (IB) quality appraised the studies using the Effective Public Health Practice Project tool^26^ for interventional or observational study designs or using an adapted version of the Consolidated Health Economic Evaluation Reporting Standards checklist^27^ for modelling studies (with questions 1, 6, 8–14, and 19–21 omitted, as these were not relevant to non-economic modelling studies). In the absence of an appropriate standardised tool for appraisal of descriptive case studies, we documented key factors that were likely to influence study quality (ie, selection or information bias, confounding risk, selective reporting, whether funding sources are detailed, and conflicts of interest). We synthesised study findings narratively. We specified in the protocol^25^ that meta-analyses would be considered for more than three papers investigating a similar intervention and quantitative primary outcome, within a similar disease context.

## Results

We identified 4033 records from database searches; two further relevant studies were identified through professional networks and one from reference lists of included studies (figure). 398 of 4036 studies were excluded as duplicates and 110 were reviewed as full text. 15 records were included and had data extracted, of which seven records were not peer-reviewed. Four of 15 records were preprint articles; one was a full-text paper and another was a poster abstract, both of which were presented at academic conferences; and one was a grey literature article. Extracted data are summarised in the tables, which detail key study characteristics, including populations, interventions, and comparators (table 1), and outcomes and key findings (table 2). Tables in the appendix further describe the key assumptions and input parameters of the modelling studies (appendix pp 5–7) and the findings and lessons learnt (appendix pp 8–10). We did not do any meta-analyses as our prespecified criteria for this were not met.

**Figure:**
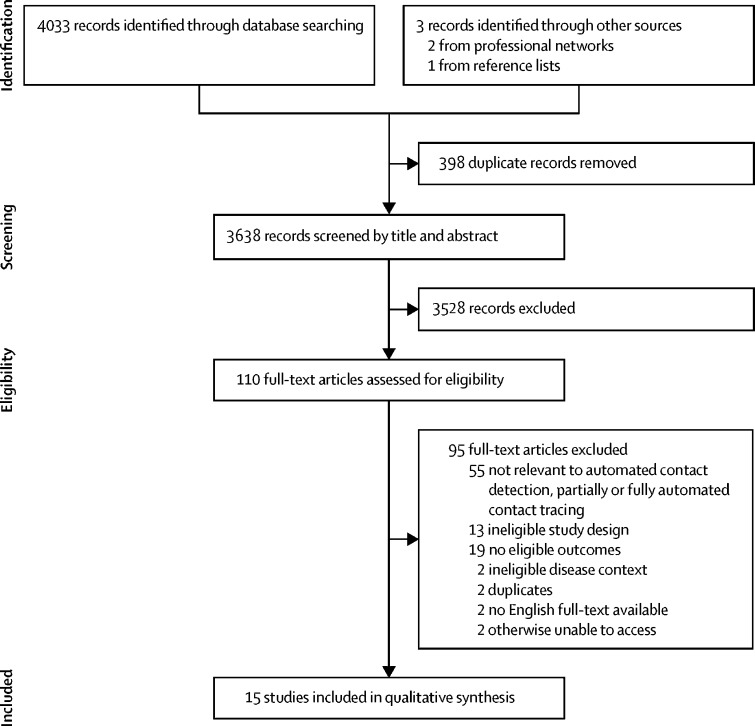
Preferred Reporting Items for Systematic Reviews and Meta-Analyses flow diagram

**Table 1:** Summary of study designs, settings, diseases under study, and characteristics of populations, interventions, and comparators

	**Study design**	**Summary; name of app or platform**	**Study setting**	**Disease under study**	**Study population**	**Number of participants or app users**	**Automated (no recall or manual data entry) or partly automated**	**Technology involved (eg, GPS, Bluetooth)**	**Intended device (eg, web browser, basic mobile phone, smartphone, multiple)**	**Contact definition used or nature of contacts (eg, recall based, proximity based)**	**Comparator**
**Fully automated contact tracing**											
Bulchandani et al (2020)^28^	Modelling study	Branching-tree mathematical model with derivations of mean-field equations for transitions to so-called digital herd immunity (ie, R_0_<1 because of automated contact tracing)	N/A	COVID-19	Hypothetical modelled population	N/A	Automated	Not specified	Not specified	Not specified	None
Ferretti et al (2020)^14^	Modelling study	Estimated the proportion of transmission from presymptomatic (from a series of 40 case pairs), asymptomatic, and symptomatic individuals and the environment; and quantified the effect of intervention (case isolation, contact tracing, and quarantine) at different delay periods and for different intervention success rates	N/A	COVID-19	Hypothetical population network and contact structures, detailed in Fraser et al (2004)^10^	N/A	Automated	Not specified	Smartphone app (standalone)	Location or proximity based	Non-automated contact tracing (comparisons based on delay to case isolation and contact quarantine)
Hinch et al (2020)^29^	Modelling study	Multiple outcomes under different scenarios involving app-based contact tracing alongside non-targeted interventions, such as lockdowns and physical distancing	UK	COVID-19	Hypothetical population network and contact structures selected to match age-stratified data reported in Mossong et al (2008)^30^	N/A	Automated	Bluetooth	Smartphone app (standalone)	Proximity based	Other non-pharmaceutical outbreak control intervention
Kim and Paul (2020)^31^	Modelling study	Effect of automated contact tracing to establish the minimum fraction of the population that needs to participate for R_0_<1	Not specified	COVID-19	Hypothetical population	N/A	Automated	Not specified	Not specified	Proximity based	Not specified
Kucharski et al (2020)^32^	Modelling study	Effect of multiple interventions (eg, app-based contact tracing, limiting daily contacts to different extents [eg, four contacts per day] in settings other than work or school, and >1 intervention in parallel) on individual-level transmission events	UK	COVID-19	Hypothetical population with contact structure based on data from the BBC Pandemic study of 40 162 UK participants^33^	N/A	Automated	Not specified	Smartphone app (standalone)	Social contacts (conversational or physical contact), as per the contact definition of the BBC Pandemic study^33^	>1 comparison (including with no contact tracing and manual contact tracing)
Xia and Lee (2020)^34^	Modelling study	Effect of automated proximity-based contact tracing; derived formulae to estimate lower and upper bounds on the minimum adoption rate required for R_0_<1	N/A	COVID-19	Hypothetical population	N/A	Automated	Proximity-based GPS data	Smartphone app or standalone wearable device	Proximity based	None
Yasaka et al (2020)^35^	Modelling study	Description of TrackCOVID, a decentralised Bluetooth-based contact-tracing app, including modelling of the population infected at different levels of uptake	N/A	COVID-19	Hypothetical population	N/A	Automated	Checkpoints based on QR codes	Smartphone app (standalone)	Face to face with manual code scanning	Compared with no contact tracing
**Partly automated contact tracing**											
Danquah et al (2019)^36^	Proof of concept study with phased introduction	Observational study regarding the introduction of the Ebola Contact Tracing app; detailed the number of contacts identified compared with previous system	Sierra Leone	Ebola	Contact tracers and contact-tracing coordinators	86 contact tracers, 26 contact-tracing coordinators	Partly automated	Manual data entry	Smartphone app (standalone)	Recall based	Other (paper-based system)
Li et al (2017)^37^	Case study	Automated identification of contacts within an inpatient setting (lists generated based on user-defined parameters)	Singapore	Multiple, including influenza A	Hospital inpatients at Changi General Hospital; system used by infection control team	Not specified	Partly automated	Real-time integration of patient movement and laboratory data	Computer-based infection control management system	Shared room, concurrent contact, and duration of contact	Non-automated contact tracing
Schafer et al (2016)^38^	Case study	App-supported contact tracing using Epi Info Viral Hemorrhagic Fever app; automation of tasks (eg, generation of daily follow-up lists); calculation of follow-up window; production of transmission chain diagrams	Seven African countries, two US states	Ebola	Used by contact tracers and contact-tracing coordinators in eight countries	Not specified	Partly automated	Manual data entry	Computer based	Recall based	Paper-based contact-tracing systems and Field Information Management System (programme developed by WHO)
Sacks et al (2015)^39^	Case study	Introduction of, use of, and lessons learned from the CommCare contact-tracing system in Guinea (compared with previous paper-based and Excel-based systems)	Guinea	Ebola	Used by contact tracers	Used by 210 contact tracers (of 366 who were trained) to collectively monitor 9162 contacts	Partly automated	Manual data entry	Smartphone app (standalone)	Recall based	Paper-based contact-tracing system (used in parallel within Guinea by other contact tracers)
Tom-Aba et al (2015)^40^	Case study	Use of Open Data Kit Collect app developed by use of Open Data Kit and FormHub to support contact tracing in Nigeria; automated alerting and SMS if any contact met the probable case definition; also refers to Ebola Sense app (supports follow-up of identified contacts, includes automated search functionality to assign contact tracers)	Nigeria	Ebola	Used by contact tracers	Not specified; all public health personnel carrying out contact tracing for Ebola cases	Partly automated	GPS (for accountability of contact tracers rather than directly within tracing efforts)	Smartphone (or tablet) app (standalone)	Recall based	Paper-based contact-tracing systems (data manually entered onto a single computer)
**Automated contact detection in a relevant disease context (without subsequent contact tracing or contact notification)**											
Aiello et al (2016; iEpi substudy)^41^	Substudy (descriptive observational study) within a cluster randomised controlled trial	iEpi substudy within a university-based trial; participants given smartphones that were used to detect other study devices and nearby Bluetooth-enabled devices to map proximity to contacts	USA	Influenza	Students aged ≥18 years	103 (iEpi substudy)	Automated contact detection only	Bluetooth	Smartphone app (standalone)	Proximity based	None
Al Qathrady et al (2016)^42^	Observational and modelling (simulation) study	Simulation of disease spread and contact tracing by use of a contact network recreated from WiFi traces; explored different approaches to prioritising investigation or follow-up of contacts at risk	Not specified	Hospital-based outbreaks	University faculty and students (contact data based on WiFi use on a university campus across six buildings)	WiFi trace data from 34 225 users of six university buildings	Automated contact detection only	WiFi network traces	Mobile devices (further detail not specified)	Proximity based	None
Voirin et al (2015)^43^	Proof of concept observational study	Pilot study that combined micro-contact data from wearable proximity sensors and virological data to investigate influenza transmission within an elderly care unit of a hospital	France	Influenza A and B	Median ages in years were 24 (doctors), 30 (nurses), and 89 (patients); proportions of women were 67% (doctors), 78% (nurses), and 73% (patients)	Total 84 (32 nurses, 15 doctors, and 37 patients)	Automated contact detection only	Radio frequency identification	Radio frequency identification proximity sensors	Proximity based	None

**GP**S=Global Positioning System. N/A=not applicable.

**Table 2:** Summary of outcomes and other key findings

	**Primary outcomes**		**Secondary outcomes**				
	Number or proportion of contacts identified (observed or required for outbreak control)	Number or proportion of subsequently diagnosed cases identified	Impact on R_0_ (or other indicators of outbreak control)	Uptake	Resource requirements or cost-effectiveness	Other ethical issues	Lessons learnt from implementation of the intervention
**Fully automated contact tracing**							
Bulchandani et al (2020)^28^	Not reported	Not reported	Estimated that approximately 90% app ownership required for epidemic control if 50% of transmission is asymptomatic but no presymptomatic transmission; multiple input parameter values assessed	Not reported	Not reported	Not reported	Not reported
Ferretti et al (2020)^14^	Multiple values; related to delay to intervention (eg, if 60% of cases are isolated, >65% of contacts need to be identified and quarantined to bring R_0_<1)	Not reported	High uptake and compliance, and decreased delays to notification (presented in a 1 day increments, from 3 days down to no delay) and quarantine improve the likelihood of reaching R_0_<1	Not reported	Not reported	Propose that such a scheme should fulfil eight requirements in order to be ethical (appendix p 8)	N/A (modelling study)
Hinch et al (2020)^29^	Baseline scenario of 3 day doubling time and 80% app uptake among smartphone owners means approximately 10–15 million people would be quarantined (at any given time, with variation over time); in addition to the population over 70 years who were assumed to practise shielding	Not reported	All app configurations showed a substantial reduction in new cases and hospital and intensive care unit admissions, and a substantial number of lives saved over a 150 day period from the start of a 35 day lockdown, increasing incrementally as uptake increases from 0% to 80% of smartphone owners; direct contact tracing (only first-order contacts quarantined) suppressed the epidemic only under optimistic epidemic growth assumptions (3·5 day doubling time, 5·0 day generation time); recursive tracing (with first-order contacts’ household members also quarantined) reached epidemic control under pessimistic assumptions but leads to only a 50% reduction in numbers quarantined compared with full lockdown; the authors estimate that test-based quarantine release would require approximately 100 000–200 000 tests per day	80% usage to reach suppression under scenarios 3 and 5 with epidemic doubling time of 3·5 days; lower uptake rates reduced incidence	Not reported	Not reported	N/A (modelling study)
Kim and Paul (2020)^31^	Not reported	Not reported	Under a range of assumptions, the percentage of the population needed to be enrolled in automated contact tracing for outbreak control (R_e_<1) was estimated (eg, 40–60% uptake required for R_e_<1, assuming a 30% mean transmission probability per contact event, if 75–95% actively confirm when they get infected; appendix p 6)	Not reported	Not reported	Not reported	N/A (modelling study)
Kucharski et al (2020)^32^	Median number of (physical and conversational) contacts successfully traced and quarantined (assuming 90% of those traced are quarantined) per case (modelled results) was 4 (mean 14) via automated contact tracing, 21 (mean 32) via manual contact tracing (acquaintances only), 28 (mean 39) with all contacts traced manually	Not reported	App-based contact tracing would require a high level of coverage to ensure R_e_<1; smaller relative reduction in R_e_ than manual contact tracing for either all contacts or acquaintances only, varying with proportion symptomatic and relative role of asymptomatic transmission; automated tracing alone was estimated to reduce R_e_ by 44%, whereas manual tracing of all contacts reduced R_e_ by 61% (assuming transmission occurs only through physical or conversational contacts that can be recalled)	Estimated 53% uptake (as an input parameter) across UK population in baseline optimistic scenario, on the basis of 75% uptake × 71% smartphone ownership	Not reported	Not reported	N/A (modelling study)
Xia and Lee (2020)^34^	Not specified; assumes 100% identification of contacts	Not reported	Assessment of the minimum uptake required to reach R_0_<1; estimated at 95–100% (if only 2–10% of cases are detected because of a large proportion being mild or asymptomatic)	Not reported	Not reported	Not reported	N/A (modelling study)
Yasaka et al (2020)^35^	Not specified	Not reported	No summary estimates presented; based on results presented graphically, an estimated 65–90% of the population would be infected at the epidemic curve peak with 0% uptake of the automated contact-tracing app, and 15–50% infected with an adoption rate of 75%	Speculated that absence of a user registration process will improve adoption rates	Suggested that because of decreased data sharing, overheads for government agencies would be minimal	Not reported	N/A (modelling study)
**Partly automated contact tracing**							
Danquah et al (2019)^36^	Mean 36 per case with app-based system; 16 per case with paper-based system	**Not specified; data suggested no identified contacts developed Ebola (however, only 69% [384 of 556] of registered contacts were visited)**	69% (384 of 556) of registered contacts for 16 confirmed cases were documented as visited under the app-based system compared with 39% (157 of 407) of contacts for nine confirmed cases for whom paper forms were returned under the paper-based system (out of 25 confirmed cases in this system; paper forms were not returned for the other 16 cases)	Not reported	Training contact tracers took 3 days; contact tracers reported that the app-based system was faster and more accurate; reduced travel time (by 5–6 h per coordinator per day); battery charging and technical support were both important	Not reported	Technical issues included poor network coverage, battery life, and quality of phones; further training on syncing data between phone and server needed; compensation and planning for phone charging, including travel to charging booths; and more refresher training for contact tracing and monitoring
Li et al (2017)^37^	Not reported	Reduced delay to intervention by the hospital infection control team by 0·5–4·0 h	Not reported	Not reported	230–476 h of contact tracing time saved per annum (baseline unclear)	Not reported	Authors stated that implementation of system took substantial time and effort from users; and workflow changes needed when specific data analytics were not available within the software
Schafer et al (2016)^38^	More than 50 000 contacts recorded on system for >100 000 cases by end of 2015	Not reported	Not reported	Widely used by contact tracers in these settings, percentage not specified	Resources required included technical expertise for training provision, data management staff who were technically skilled and trained (the authors noted that these skills were often scarce locally), and time and expertise required for set-up and maintenance of the database and network; network connectivity and electricity supply were also important resources, and recurrent outages made data entry and transmission challenging	Not reported	Successful use of app required organised flow of contact information between data managers and contact tracers, and a concerted effort to use the app (often not the case); Epi Info Viral Hemorrhagic Fever app designed to require minimal IT support but multiuser data entry expanded complexity and support requirements (minimal, if any, IT support was available); accommodating many countries’ needs within one software was challenging, particularly as it was not possible to customise the software for each country
Sacks et al (2015)^39^	210 contact tracers monitoring 9162 contacts	Not reported	Not reported	210 of 366 who were trained on CommCare actively used it	Total time to establish programme was 10–13 weeks (training contact tracers took 2–3 days); smartphones, SIM cards, 500 MB data per month, and solar phone chargers donated for staff use; need for technical support	Focused on the ethical issues around adopting new technology during complex emergency; authors argue this disruption might be required, but that feasibility and risks should be carefully considered and, where implemented, accompanied with close managerial oversight	Availability and cost of expertise for specific software used; recruitment of local technology expert youth volunteers helped to accelerate phone configuration; actual use of data by government staff members to inform action was low; clearer initial standards (for contact-tracing protocols and metrics) could have accelerated the design process
Tom-Aba et al (2015)^40^	Not reported	Not reported	Reduced delay to evacuation of symptomatic contacts from their homes to an isolation facility from 3–6 h to 1 h, and the proportion of contacts followed up increased from a variable baseline of between 90% and 99% (before the partly automated system was introduced) to consistently 100% afterwards, with associated benefits for outbreak control	Not reported	Costs of android phones, tablets, laptops, data plans, and high-speed internet; time costs of trained personnel	Not reported	Not reported
**Automated contact detection in a relevant disease context (without subsequent contact tracing or contact notification)**							
Aiello et al (2016)^41^	453 281 total Bluetooth contacts between iEpi substudy smartphones only (62·5 contacts per phone per day) and 1 591 741 with other devices (219·4 contacts per phone per day) over 78 days	N/A	Not reported	Not reported	Availability and training of a large study staff (size not specified); system required mapping, debugging, data cleaning, and verification	Not reported	95% (281 of 295) of participants who completed the exit survey reported joining the study because of the cash incentive
Al Qathrady et al (2016)^42^	353 458 encounter records from 34 225 users in 1 week	Not reported	All contacts within the simulated institutional outbreak could be traced on the basis of one infectious case having been identified (for an infection with a latent period of 1 day and infectious period of 2 days); further detail not provided	Not reported	Not reported	Not reported	Accuracy of the contact-tracing system in some buildings decreased with coverage, but increased in others, because of differences in the encounter patterns within each building, and the source node chosen
Voirin et al (2015)^43^	18 765 contact events recorded among 84 individuals over 11 days (cumulative duration 251 h)	N/A	Not reported	Not reported	Not reported	Not reported	Most contacts between nurses or between nurses and a patient; influenza transmission is difficult to predict from contact data alone

Additional details can be found in the appendix (appendix pp 8–10). N/A=not applicable. R_0_=basic reproduction number. R_e_=effective reproduction number.

Findings from the included studies are detailed here in three categories: seven studies that addressed automated contact tracing directly (all modelling studies that focused on COVID-19);^14^, ^28^, ^29^, ^31^, ^32^, ^34^, ^35^ five descriptive observational or case studies of partly automated contact tracing (four studies related to Ebola virus disease^36^, ^38^, ^39^, ^40^ and one study of a hospital infection control system);^37^ and, in a post-hoc definition, three studies of automated contact detection within a relevant disease context but without subsequent tracing or contact notification.^41^, ^42^, ^43^ No study assessed ethical considerations from an empirical perspective, although such considerations were discussed in theoretical terms by two studies (table 2; appendix pp 8–10).^14^, ^39^

### Studies of automated contact tracing in COVID-19

We identified seven studies of automated contact tracing; all of which were mathematical modelling studies, with varied assumptions (appendix pp 5–7). Five of these seven studies addressed smartphone apps specifically,^14^, ^29^, ^32^, ^34^, ^35^ alongside other wearable devices in one of these studies.^34^ The other two studies related to an unspecified type of device carried by users.^28^, ^31^ No studies contained data for our primary outcomes with the same definition given in our protocol (number or proportion of contacts, and of contacts that go on to become infected, identified); however, two of the seven modelling studies provided data of a similar and relevant nature, in the form of the estimated number of contacts quarantined.^29^, ^32^

One modelling study of control measures for COVID-19 in the UK estimated that a median of four contacts per case (mean 14) would be quarantined under automated contact tracing, compared with 28 (mean 39) with all contacts traced manually, assuming 90% adherence to quarantine.^32^ Another modelling study of COVID-19 in the UK assumed 100% initial adherence to quarantine and 80% uptake among smartphone owners.^29^ This study estimated that approximately 10–15 million people would be quarantined (cumulatively and at any given time, alongside the population older than 70 years, who were assumed to be shielding) but did not present the number of contacts identified per case. Three studies described an approximately quadratic relationship between population uptake of an automated contact-tracing tool (such as an app) and associated reductions in transmission, under various simplifying assumptions.^28^, ^31^, ^32^ This relationship would mean that an 80% uptake might enable notification of approximately 64% of the contacts who would be notified in an optimal contact-tracing system; whereas, with 50% uptake the corresponding figure is 25% (appendix p 8).^31^

Only Kucharski and colleagues^32^ directly compared the modelled effects of automated contact tracing on R_0_ or R_e_ with those of manual contact tracing. Under what they termed optimistic assumptions, including 75% uptake among smartphone owners and assuming equal maximum delays to quarantine of contacts under automated and manual scenarios, Kucharski and colleagues^32^ estimated that automated tracing alone reduced R_e_ by 44%, whereas manual tracing of all contacts reduced R_e_ by 61% (this study uses a contact matrix based on self-reported physical or conversational contacts from another study,^33^ so effectively assumes no transmission from other kinds of contact). Hinch and colleagues^29^ did not compare automated and manual contact tracing or report the effects on R_e_. Both studies^29^, ^32^ reported that suppressing the COVID-19 outbreak required concurrent measures (eg, shielding vulnerable groups,^29^ remote working, and limiting the number of contacts per day to fewer than 10 outside of work and school)^32^ alongside automated contact tracing. Most of the scenarios that were modelled by Hinch and colleagues^29^ did not result in containment (which is approximately equivalent to bringing R_e_<1), except when quarantining all household members of contacts who had direct contact with a case (recursive contact tracing).^24^

Two other modelling studies of automated contact tracing for COVID-19 reported similar findings, with high uptake required to substantially suppress transmission: one study estimated a population-wide uptake of 75–95%,^28^ and another 90–95%,^34^ to reduce R_e_ to less than 1. Several studies found that, even at uptake levels less than those required to reach R_e_ less than 1, increasing rates of uptake were associated with decreased incidence rates of COVID-19.^28^, ^29^, ^35^ Another modelling study emphasised the importance of timely quarantine of contacts for effectiveness with respect to outbreak control, showing that, in contact tracing for COVID-19, the proportion of contacts needing to be quarantined to reach R_e_ less than 1 increased markedly as delays between exposure to a case and quarantine increased from 0 days to 3 days.^14^ The authors concluded from these results that an app capable of instantaneous contact tracing and notification could help to control the epidemic.^14^

Regarding resource requirements, one study estimated that between 100 000 and 200 000 tests per day would be required for test-based quarantine release in the UK;^29^ another estimated 30–50 tests required per case detected.^32^ No other secondary outcome data were reported for studies of automated contact tracing in COVID-19.

### Studies of partly automated contact tracing

We identified a total of five studies of partly automated contact tracing, which all automated some processes within systems involving human contact tracers or infection control staff. One study in Singapore profiled a system based in a hospital that automatically alerts staff to new infections by target organisms and generates contact lists by use of user-defined parameters (eg, having shared a room, concurrent contact, and duration of contact).^37^ Four studies focused on software apps used to manage Ebola virus disease outbreaks.^36^, ^38^, ^39^, ^40^

Three of these studies reported data that were relevant to our primary outcomes; in one study, a mean of 36 contacts per person with Ebola virus disease was recorded when contact tracers used an app (Ebola Contact Tracing app)^36^ compared with 16 contacts per person with the disease in the pre-existing manual contact-tracing system that used paper forms and Microsoft Excel. In a second study, more than 100 000 investigated cases of disease and more than 50 000 contacts were recorded in the Epi Info Viral Hemorrhagic Fever app by contact tracers across seven African countries and two US states by the end of 2015; the reason was unclear for this apparent low ratio of approximately only 0·5 contacts per case recorded.^38^ A third study of the CommCare app, a partly automated app with algorithm-based features that supports decisions (eg, prompting referral for testing following the entry of data indicating that a contact developed symptoms) and updates a data visualisation dashboard automatically every hour, reported 9162 contacts, but the number of people who became infected in these contacts was unspecified.^39^ No other primary outcomes were reported in these studies.

No study investigated the effects of partly automated contact tracing on R_0_ or R_e_; however, the completeness of follow-up of contacts (ie, the proportion of identified or recorded contacts who were followed up) was greater than in the previously used manual comparator systems in two studies.^36^, ^40^ For example, in one study in Sierra Leone, 69% (384 of 556) of contacts for confirmed cases were documented as visited under the app-based system for 16 confirmed cases compared with 39% (157 of 407) of contacts for the nine confirmed cases for whom paper forms were returned.^36^ In another study in Nigeria,^40^ the proportion of contacts followed up increased from approximately 90% to 99%, with variation over time, to 100%, consistently, after introduction of a partly automated contact-tracing system. Two studies of partly automated systems reported modest improvements in intervention timeliness (eg, quarantine or isolation): one of these studies reported decreased delays to quarantine of symptomatic contacts (by 2·0–5·0 h)^40^ and another^37^ reported decreased delays to review and action by a hospital infection control team in an inpatient setting (by 0·5–4·0 h per patient).

Three studies detailed the hardware, software, and supporting infrastructure requirements of partly automated contact-tracing systems;^36^, ^39^, ^40^ these included smartphones, tablets, laptops, SIM cards, data plans, high-speed internet, and phone battery charging. No study of partly automated contact tracing provided cost information and only one study detailed the duration of implementation (10–13 weeks).^39^ One study reported that approximately 230–476 h per year of contact-tracing work was saved by a partly automated infection control management system in a Singaporean hospital.^37^ In another study, contact tracers reported that the app-based system was faster and more accurate than a paper-based system and eliminated substantial travel time (5–6 h per coordinator per day).^36^

Technical support needs, including for training, were a recurrent theme. For example, one study of the Epi Info Viral Hemorrhagic Fever app in eight countries stated that training was often provided by staff who “had received only minimal training themselves” leading to “inefficient and incorrect use”;^38^ technical expertise was emphasised as an important but scarce resource in two other studies.^36^, ^40^ One study reported that training contact tracers took 2–3 days^39^ and another study stated that training took 3 days.^36^ Other lessons learnt included the importance of reliable internet and electricity infrastructure,^36^, ^38^ and the value of customising systems on the basis of local priorities.^38^

### Other studies relevant to automated contact tracing

We found three studies of contact detection in a relevant disease context but without subsequent tracing or contact notification: one studied students' smartphone contact patterns,^41^ another integrated radiofrequency contact and virological data in a hospital setting,^43^ and another used WiFi traces on a university campus to model a hypothetical epidemic.^42^

None of these studies detailed a primary outcome precisely as specified; however, participants in the iEpi substudy^41^ had a mean average of 219 contacts per phone per day, while another study observed 18 765 contact events among 84 participants over 11 days (but only four influenza transmission events).^43^ Regarding resource requirements, one study referred to the need for the availability and training of a large study staff.^41^ Lessons learnt are detailed in table 2, with further detail in the appendix (appendix pp 8–10). No other secondary outcomes were detailed.

### Quality assessment

Study quality was varied and quality assessments are detailed in the appendix (appendix pp 11–16). The quality of studies of automated contact tracing in COVID-19 and partly automated contact tracing for other infectious diseases, such as Ebola virus disease and influenza, was generally limited by their observational and often descriptive nature, without prespecified protocols (except in one article, in which the protocol was modified during the study^36^). Many studies were subject to possible confounding, selection bias, and selective reporting. Among the modelling studies, some included detailed methods, did sensitivity analyses, and provided their model code.^14^, ^29^, ^32^ Others sometimes provided little justification of the model structures or assumptions used, with assumptions not always based on the relevant available evidence. The epidemiological parameters selected in one study were not chosen to represent SARS-CoV-2, so the findings might not be relevant in the context of controlling COVID-19.^42^ No study modelled heterogeneous levels of smartphone usage or app uptake (eg, by income level or age), however, two studies assumed that no one younger or older than a specific age threshold (ie, younger than 10 years and older than 70 or 80 years) used smartphones. Four modelling studies did not account for uncertainties or they did few sensitivity analyses.^28^, ^31^, ^34^, ^35^

## Discussion

We did not identify any epidemiological studies comparing automated with manual contact-tracing systems and their effectiveness in identifying or notifying contacts, either for COVID-19 or another included disease. As a result, manual contact tracing on a large scale is still likely to be required in most contexts, and there is a clear need for further research to strengthen the evidence base for automated contact tracing. Future research should assess the empirical effects on disease transmission and the effects of technical aspects of contact-tracing apps on the uptake and effectiveness, ethical and equity considerations, and interactions with manual contact-tracing systems. We did identify several observational and case studies of partly automated contact-tracing systems in other disease contexts.

Taken together, the modelling studies that we identified showed that the effectiveness of automated contact tracing in reducing disease transmission depends on both population uptake (eg, of contact-tracing apps) and timeliness of intervention (eg, quarantining contacts).^14^ As with manual contact tracing, automated contact tracing also relies on accurate and reliable identification of encounters during which transmission occurs.

The effectiveness of contact tracing depends on the disease context; system factors, such as the timeliness of case identification and contact notification, contact tracers' expertise, and the case and contact definitions used; and context-dependent social and behavioural factors such as self-reporting rates and quarantine adherence.^8^, ^10^, ^12^, ^44^ Many of these points apply to both manual and automated contact tracing; key differences with automated contact tracing include the possibility of minimising the “recall problem”^34^ of manual contact tracing, thus enabling tracing of contacts who are at high risk, allowing faster contact notification and quarantine, and potentially enabling systems to scale up faster and with fewer resources than with a manual approach. One study compared the expected modelled effectiveness of manual contact tracing with automated approaches and showed that manual contact tracing is able to reduce R_e_ by more than automated contact tracing is able to.^32^ Another modelling study investigated the effect of faster notification,^14^ but no other study examined the potential effect of these three factors specifically.

Uptake is particularly important, since both the people with the infection and their contacts need to have and use a system for it to have any effect. This leads to a quadratic relationship (under simplifying assumptions), such that effectiveness drops off steeply as participation falls. Even under optimistic assumptions (eg, 75–80% app uptake among smartphone owners in a context with high smartphone ownership and 90–100% adherence to quarantine), automated contact tracing appears unlikely to control the spread of COVID-19 without concurrent measures;^29^, ^32^, ^34^ something that is even more the case in settings with low smartphone ownership.^28^

Our primary outcomes, regarding the numbers and proportions of contacts identified (including of those who become infected), are a key gap in current evidence and important metrics for evaluation. These metrics are related to issues of false positive events (encounters where viral transmission did not occur but that result in a contact being traced) and false negative events (actual transmission events that, despite app or tool use by the relevant case and contact, do not result in a contact being traced). These events are potentially problematic in both automated and manual contact-tracing systems—albeit for slightly different reasons—and there are trade-offs between them.^13^ False positives cause problems because they are likely to reduce uptake and quarantine adherence, and because of the adverse psychological effects and wider harms of quarantine,^4^, ^44^ and false negatives are a missed opportunity to prevent onward transmission. However, how the risks of each event compare between manual and automated contact-tracing systems was not well evaluated by any of the included studies.

Several authors have emphasised the importance of the technical dimensions of automated contact tracing,^21^, ^45^, ^46^ such as compatibility with older smartphones and whether an app works while running in the background,^20^ alongside the fact that they cannot account for risk-modifying factors effectively (eg, use of personal protective equipment; separation by screens or walls—where a Bluetooth signal can pass through but a virus cannot; or ventilation levels). These technical questions have implications for the frequency of false positive and false negative events; as one study noted, “the accuracy with which Bluetooth low-energy signatures can be converted to useful proxies of transmission risk is currently uncertain”.^29^ The potential effect of such factors is not examined by the included studies and addressing this gap will require data from real world settings.

The integration and effect of manual and automated systems that are run in parallel were not examined by the included studies. The extent of presymptomatic transmission might be substantial in COVID-19,^47^, ^48^ making the timeliness of quarantine critical.^10^, ^14^ However, the timeliness of automated versus manual contact-tracing systems is unknown and will also be influenced by whether notification is based on symptoms or tests, and therefore, by test turnaround times.^29^ Two partly automated systems that were studied appeared to reduce delays to quarantine by a modest amount.^37^, ^40^ Additionally, whether quarantine adherence differs between automated and manual systems is unknown. Automated notification might be psychologically different from receiving a telephone call from a human contact tracer, who can give detailed information about what action to take and why, check understanding, and address questions or concerns.^56^ A previous review found adherence to quarantine to be extremely varied and influenced by multiple factors, including risk perception and social and financial protections.^42^

Academics have warned of the risks that automated contact tracing could pose, including a so-called mission creep towards unprecedented surveillance and eroded public trust, should data be misused or hacked.^49^ Privacy and information governance are also highly important within manual contact-tracing systems, but given the substantially larger amounts of personal data (including colocation or location data, or both) that could be collected and processed in automated systems, they are particularly important considerations in this context. However, a detailed consideration of privacy and information governance is beyond the scope of our systematic review and these points are discussed in depth elsewhere.^23^, ^50^, ^51^ Trade-offs between privacy and utility are also discussed elsewhere;^17^, ^23^ these factors might vary between system architectures, particularly centralised systems, which involve data being uploaded to a central server, and decentralised systems, which preserve privacy more strongly, keeping colocation data on users' phones.^52^

Optimising risk thresholds to simultaneously minimise transmission risk and the number of people quarantined is a key challenge for any contact-tracing system,^12^ particularly in view of the adverse psychological effects and wider harms of quarantine.^4^, ^44^ However, setting thresholds that minimise both false positive and false negative events relies on gathering and analysing large datasets of high quality.^52^

Decentralised automated contact-tracing systems benefit from Apple and Google's support, meaning that interoperability between countries with such apps is likely to be more straightforward than between countries that use centralised systems.^20^ However, a study reported that centralised systems assess transmission risk more accurately (reducing the number of people quarantined), enable better optimisation, are less susceptible to false reports, and are more readily evaluated.^52^

Wider concerns around digital exclusion and broader ethical concerns have been emphasised elsewhere,^22^, ^53^ including in the Ada Lovelace Institute rapid review,^21^ but are not currently well quantified. Some populations that are particularly vulnerable to the health impacts of COVID-19 (eg, older adults, people who are homeless, and socioeconomically deprived populations) are also less likely to own a smartphone,^17^, ^22^, ^54^, ^55^ potentially amplifying their risks because contact-tracing apps could—for similar reasons—be less likely to reduce transmission within their social circles.^33^ Such challenges are more acute in low-income countries than in high-income countries.^28^

Key questions that policy makers should consider before implementing an automated contact-tracing system, and that future research should seek to answer, include whether concerns around public acceptability and privacy have been adequately addressed, with appropriate public consultation; how an automated system will be integrated with other contact-tracing and disease control strategies, in consultation with public health experts; and, perhaps most importantly, whether it is likely to be effective, cost-effective, and equitable in that context. Where automated contact-tracing systems are deployed, they should be evaluated rigorously, including through large-scale prospective studies of effectiveness, technical and equity dimensions, and qualitative studies to improve the understanding of key social and behavioural dimensions of app use and adherence.^11^, ^52^

The strengths of this systematic review include the comprehensive search strategy and prespecified eligibility criteria and screening process. With its focus on outbreak control, this systematic review also addresses timely, policy-relevant questions. However, there are some limitations. We assessed all studies for quality but were unable to do a meta-analysis and a formal assessment of publication bias. There was a scarcity of eligible empirical studies of fully automated contact tracing. There were limitations of the approaches used by modelling studies (eg, a number of them did not account for time delays or presymptomatic transmission, did not facilitate modelling of depletion of susceptible individuals, and had poorly evidenced assumptions, for example, a high quarantine adherence), and a paucity of evidence related to ethical concerns or cost-effectiveness. Another limitation of this systematic review is that four of 15 studies included in the analysis were preprints,^28^, ^31^, ^32^, ^34^ two records were from conference proceedings (one poster abstract^37^ and one full text^42^), and one article was considered to be grey literature,^29^ none of which were peer-reviewed. The modelling studies reflect substantial uncertainty; for example, if environmental transmission of SARS-CoV-2 occurs frequently, this would undermine the validity of their results, since proximity-based tracing apps detect colocation but do not detect intermediary contact with potentially contaminated surfaces or fomites.^46^ These kinds of uncertainties, related both to various aspects of the transmission and epidemiology of SARS-CoV-2 and to human behaviour under new, untested scenarios, make it difficult to objectively appraise how realistic the assumptions (and therefore the results) of the modelling studies are. Additionally, our systematic review was limited to studies published in English because of short timescales.

Given the substantial uncertainties about the effectiveness of automated contact-tracing systems, manual contact tracing on a large scale is likely to be required to control COVID-19, alongside measures such as remote working by a proportion of the population and physical distancing. There is potential for manual contact tracing to operate alongside, and be supported by, automated approaches; moderate uptake of automated systems could contribute to reducing transmission and offset some of the work of manual contact tracing. However, the potential benefits of automated approaches should be weighed against the implementation costs and broader risks, for example, around equity and privacy considerations. Decision makers should use all of the available evidence to ensure that contact-tracing systems used to control COVID-19 (which might have both manual and automated components) are as effective, equitable, and privacy preserving as possible, and should consult the public with regard to potential trade-offs between these policy objectives. Decision makers should also ensure that, where automated contact-tracing systems are implemented, they are thoroughly evaluated and their use is within the context of comprehensive, integrated outbreak prevention and response plans.
