# Supplementary material for: Automated and partly automated contact tracing: a systematic review to inform the control of COVID-19
Source: Lancet Digit Health. 2020 Aug 19;2(11):e607–21. doi: 10.1016/S2589-7500(20)30184-9 (PMC7438082; doi:10.1016/S2589-7500(20)30184-9)
Supplement: Supplementary appendix [file mmc1.pdf]

THE LANCET
Digital Health

Supplementary appendix

This appendix formed part of the original submission and has been peer reviewed. We post it as supplied by the authors.

Supplement to: Braithwaite I, Callender T, Bullock M, Aldridge RW. Automated and partly automated contact tracing: a systematic review to inform the control of COVID-19. *Lancet Digital Health* 2020; published online Aug 19. https://doi.org/10.1016/S2589-7500(20)30184-9.

Automated and partly automated contact tracing: a systematic review to inform the control of COVID-19

Appendix

Isobel Braithwaite[1], Tom Callender[2], Miriam Bullock[3], Robert W Aldridge[1]

[1] UCL Public Health Data Science Research Group, Institute of Health Informatics, University College London, London, UK

[2] Department of Applied Health Research, University College London, London, UK

[3] UCL Collaborative Centre for Inclusion Health, University College London, London UK

Correspondence:

Dr Isobel Braithwaite,
Institute of Health Informatics,
University College London,
222 Euston Road,
London, NW1 2DA
Isobel.Braithwaite.13@ucl.ac.uk

Table of Contents

Section	Page Number
Details of search strategies	1
PubMed search strategy	1
Embase search strategy	1
OVID Global Health search strategy	2
MedRXiv, bioRXiv, arXiv search strategies	3
Cochrane Library search strategy	4
EBSCO COVID Information Portal search strategy	4
Google Advanced search strategy	4
Table S1: Relevant (base case) assumptions and scenarios modelled or key sensitivity analyses conducted (modelling studies only)	5
Table S2: Detailed lessons learned and other key findings	8
Table S3 – Effective Public Health Practice Tool Quality Assessment	11
Table S4 – Quality assessment using adapted CHEERS Checklist	13
Table S5 Quality assessment notes – case studies only	16
Table S6: Studies selected for level 2 (full-text) screening which have no full-text available in English	17
References	18

Details of search strategies:

(All searches were limited by the dates 01/01/2000 to 14/04/2020)

PubMed search strategy:

((digital*[Title/Abstract]) OR ("digital divide"[MeSH Major Topic]) OR (digital[MeSH Subheading]) OR (mobile phone[MeSH Terms]) OR (cell phone[MeSH Terms]) OR ("smartphone"[MeSH Terms]) OR ("internet"[MeSH Terms]) OR (digital*[MeSH Terms]) OR (automat*[MeSH Terms]) OR (technology[MeSH Terms]) OR (tech[MeSH Terms]) OR (electronic*[MeSH Terms]) OR (app[MeSH Terms]) OR (application[MeSH Terms]) OR (smart-phone*[MeSH Terms]) OR (phone*[MeSH Terms]) OR (online*[MeSH Terms]) OR (m-Health[MeSH Terms]) OR (mHealth[MeSH Terms]) OR (e-Health[MeSH Terms]) OR (eHealth[MeSH Terms]) OR (telehealth*[MeSH Terms]) OR (tele-health*[MeSH Terms]) OR (digital*[Title/Abstract] OR automat*[Title/Abstract] OR technology[Title/Abstract] OR tech[Title/Abstract] OR electronic*[Title/Abstract] OR app[Title/Abstract] OR application[Title/Abstract] OR smartphone*[Title/Abstract] OR smart-phone*[Title/Abstract] OR mobile*[Title/Abstract] OR phone*[Title/Abstract] OR online*[Title/Abstract] OR internet*[Title/Abstract] OR m-Health[Title/Abstract] OR mHealth[Title/Abstract] OR eHealth[Title/Abstract] OR eHealth[Title/Abstract] OR telehealth*[Title/Abstract] OR tele-health*[Title/Abstract]))

AND

(("contact trac*"[Title/Abstract]) OR ("contact-trac*"[Title/Abstract]) OR ("outbreak control"[Title/Abstract]) OR ("health protection"[Title/Abstract]) OR (“outbreak prevention”[Title/Abstract]) OR (“infectious disease control”[Title/Abstract]) OR ("communicable disease control”[Title/Abstract]) OR (contact tracing[MeSH Terms]) OR (communicable disease contact tracing[MeSH Terms]) OR (infectious disease contact tracing[MeSH Terms]) OR (disease outbreak, infectious[MeSH Terms]) OR ("outbreak control"[MeSH Terms]) OR ("outbreak prevention"[MeSH Terms]) OR (infectious disease control[MeSH Terms]) OR (communicable disease control[MeSH Terms]) OR (contact tracing[Supplementary Concept]))

AND

((COVID*[Title/Abstract] OR SARS-CoV-2[Title/Abstract] OR novel coronavirus[Title/Abstract] OR nCoV-2019[Title/Abstract] OR MERS[Title/Abstract] OR MERSCoV[Title/Abstract] OR SARS[Title/Abstract] OR SARS-CoV[Title/Abstract] OR influenza*[Title/Abstract] OR H1N1[Title/Abstract] OR "swine $flu*"[Title/Abstract] OR H5N1[Title/Abstract] OR "avian flu*"[Title/Abstract] OR "avian influenza*"[Title/Abstract] OR "bird $flu*"[Title/Abstract] OR pandemic*[Title/Abstract] OR ebola[Title/Abstract] OR ebola hemorrhagic fever[Title/Abstract] OR ebolavirus[Title/Abstract]) OR ("pandemics"[MeSH Terms]) OR (ebola hemorrhagic fever[MeSH Terms]) OR (ebolavirus[MeSH Terms]) OR (coronavirus, sars[MeSH Terms]) OR (coronavirus, sars related[MeSH Terms]) OR (coronavirus, sars associated[MeSH Terms]) OR ("middle east respiratory syndrome coronavirus"[MeSH Terms]) OR (COVID-19[Title/Abstract])) OR (MERS[MeSH Terms]))

Embase search strategy:

1 exp pandemic influenza/ or exp pandemic/ or pandemic.mp.

2 coronavirus infection/ or exp middle east respiratory syndrome/ or exp severe acute respiratory syndrome/
3 COVID-19.mp.
4 SARS.mp. or exp severe acute respiratory syndrome/
5 MERS.mp.
6 bird flu.mp. or exp avian influenza/
7 swine flu.mp. or exp swine influenza/
8 H1N1.mp. or exp "Influenza A virus (H1N1)"/
9 H5N1.mp. or exp "Influenza A virus (H5N1)"/
10 ebola.mp. or exp Ebola hemorrhagic fever/
11 exp Zaire ebolavirus/ or exp Ebolavirus/ or ebolavirus.mp.
12 SARS-CoV.mp. or exp SARS coronavirus/
13 MERS-CoV.mp. or exp Middle East respiratory syndrome coronavirus/
14 1 or 2 or 3 or 4 or 5 or 6 or 7 or 8 or 9 or 10 or 11 or 12 or 13
15 exp communicable disease control/ or disease control/ or disease notification/ or exp infection control/ or exp isolation/ or exp quarantine/
16 outbreak prevention.mp.
17 health protection.mp.
18 contact tracing.mp. or exp contact examination/
19 case finding.mp. or exp case finding/
20 exp disease notification/
21 15 or 16 or 17 or 18 or 19 or 20
22 digital.mp.
23 smartphone.mp. or exp mobile phone/ or exp smartphone/ or exp computer program/ or exp telemedicine/
24 technology/ or exp medical technology/ or exp information technology/ or technology.mp.
25 app.mp. or exp mobile application/
26 tech.mp. or exp technology/
27 internet.mp. or exp Internet/
28 e$health.mp. or telehealth/ 29 m$Health.mp.
30 tele$health.mp. or exp telehealth/
31 22 or 23 or 24 or 25 or 26 or 27 or 28 or 29 or 30
32 exp influenza/ or influenza.mp.
33 14 or 32
34 21 and 31 and 33
35 " limit 34 to yr=""2000 -Current"" "

OVID Global Health search strategy:

3. digital.mp. or exp digital technology/
4. smartphone.mp. or exp mobile telephones/

5. internet.mp. or exp internet/
6. online.mp. or (on line or information retrieval).sh.
7. technology/ or digital technology/ or application/
8. m-health.mp. or mobile telephones.sh. or telemedicine.sh.
9. eHealth.mp. or computers.sh.
10. information technology.mp. or exp information technology/
11. exp automation/
12. automat*.mp.
13. exp mobile equipment/ or mobile.mp.
14. phone.mp.
15. app.mp.
16. 3 or 4 or 5 or 6 or 7 or 8 or 9 or 10 or 11 or 12 or 13 or 14 or 15
17. contact tracing.mp. or exp contact tracing/
18. contact trac*.mp.
19. disease control/ or exp contact tracing/ or exp control programmes/ or exp disease prevention/ or exp health protection/ or exp infection control/
20. exp outbreaks/
21. outbreak control.mp.
22. infectious disease control.mp.
23. communicable disease control.mp.
24. health protection.mp. or exp health protection/
25. Severe acute respiratory syndrome coronavirus.od. or COVID.mp. or severe acute respiratory syndrome.sh. or Middle East.gl. or Middle East respiratory syndrome coronavirus.od.
26. pandemic.mp. or exp pandemics/
27. influenza/ or exp avian influenza A viruses/ or exp avian influenza/ or exp swine influenza viruses/ or exp avian influenza viruses/ or exp swine influenza/ or exp swine influenza A viruses/
28. COVID-19.mp.
29. 17 or 18 or 19 or 20 or 21 or 22 or 23 or 24
30. 25 or 26 or 27 or 28
31. 16 and 29 and 30
32. from 31 keep 1-486 (01/01/2000 to 14/4/2020)

MedRXiv, bioRXiv, arXiv search strategies:

After a number of trial searches, medRXiv, BioRXiv and arXiv were searched using only the term ‘contact tracing’

Cochrane Library search strategy:

The search terms were adapted after trial searches. It was finally searched by ‘contact tracing’ (including subsidiary MeSH trees), and by ‘(smartphone OR mobile applications OR automation) AND (contact tracing OR disease outbreaks OR infectious disease transmission)’, alongside all linked MeSH terms (as below); the same search was also conducted including the term ‘digital’ within the group of (#1 OR #2 OR #3):

#1 MeSH descriptor: [Smartphone] explode all trees
#2 MeSH descriptor: [Mobile Applications] explode all trees
#3 MeSH descriptor: [Automation] explode all trees
#4 MeSH descriptor: [Contact Tracing] explode all trees
#5 MeSH descriptor: [Disease Outbreaks] explode all trees
#6 MeSH descriptor: [Disease Transmission, Infectious] explode all trees
#7 (#1 OR #2 OR #3) AND (#4 OR #5 OR #6)

EBSCO COVID Information Portal search strategy:

No Boolean operator functionality; separate searches therefore conducted using the search terms ‘app’, ‘automated’, ‘digital’ and ‘contact tracing’

Google Advanced search strategy:

(automat* OR digital* OR technology OR electronic* OR app OR smartphone* OR mobile* OR cellphone* OR online* OR internet* OR mHealth OR eHealth OR telehealth*)
AND
("contact tracing" OR "contact trace" OR "outbreak control" OR "communicable disease control")
AND
(COVID-19 OR SARS-CoV-2 OR nCoV-2019 OR MERS OR SARS OR H1N1 OR H5N1 OR swine flu OR avian flu OR bird flu OR pandemic OR ebola OR ebolavirus)

Table S1: Relevant (base case) assumptions and scenarios modelled or key sensitivity analyses conducted (modelling studies only)

Reference	Description of model	Key relevant assumptions (base case/central scenarios)	Scenarios modelled and key sensitivity analyses conducted
Al Qathrady et al. 2016	Stochastic SEIR (susceptible, exposed, infectious, recovered) disease simulation model using contact network based on WiFi trace data.	• Outbreak seeded with 1 infection<br>• Transmission probability = 0.005 per second in proximity (minimum distance not specified); all close contact events have an equal transmission risk per second.<br>• Latent period = 1 day<br>• Infectious period = 2 days<br>• Infection does not change behaviour/contact patterns	Comparison of random-selection of at-risk contacts for follow-up/testing with two alternative algorithms; 'mostly-named' (prioritisation of contacts based on the number of times they are identified as at risk by index cases) and 'encounter infection probability' (based on cumulative probability of infection according to duration of all encounters with infected individuals)
Bulchandani et al. 2020	Branching-process model with simulations performed on 10,000 nodes (each representing a contact event between two or more individuals) with 100 initial infections	• Branching process generational model, simulating an epidemic spreading through a population of people who are susceptible to the disease, in discrete time. In this model, discrete time is considered as generational steps: Individuals infected at the beginning of each generation are assumed not to infect anyone else after that generation has elapsed.<br>• $R_0 = 3$,<br>• $R_{symptomatic} = 1$<br>• θ (fraction of asymptomatic cases) = 0.2 − 0.5<br>• 4000 simulation runs of 10,000 people, starting with 100 initial infections and running over 20 generations of infection.<br>• Assumes recursive contact notification, defined in this paper as including contacts from previous model generations (who may have given the confirmed case their infection) and their recent contacts.	60-90% app ownership/use in 10% increments (with θ, the fraction of asymptomatic cases, fixed at 0.5) 20 to 50% asymptomatic transmission<br>$R_{symptomatic}$ (the average number of people in a susceptible population who would be infected by each infected symptomatic person due to presymptomatic transmission only, i.e. if isolation occurred immediately on development of symptoms) = 0, 1 or 2
Ferretti et al. 2020	Mathematical model of infectiousness to quantify pre-, a- and symptomatic transmission, as well as environmental contributions to transmission risk; estimation of the probability of success of contact tracing	• 40% of COVID-19 infections are asymptomatic; these are 10% as infectious as symptomatic infections<br>• (Initial) epidemic doubling time = 5.0 days (95% C.I. 4.2 to 6.4)<br>• Incubation period – lognormal distribution with a mean of 5.2 days and a standard deviation of 1.44 days.<br>• R0 = 2.0 (comprised of: 0.9 from pre-symptomatic transmission, 0.8 from symptomatic transmission, 0.2 from environmentally mediated, 0.1 from asymptomatic transmission)<br>• No delay between case confirmation and notification of contacts - total delay for the contact quarantine process is the sum of the delay from an individual's symptom onset to their confirmation as a case, plus that for notified contacts to enter quarantine after being notified	Varying success rate of instant isolation of symptomatic cases and the success rate of instant contact tracing (both from 10-100%, presented graphically).<br>N.B. The success rate of case isolation "can be thought of either as the fraction of symptomatic individuals isolated, assuming perfect prevention of transmission on isolation, or the degree to which infectiousness of symptomatic individuals is reduced assuming all of them are isolated". The success rate of contact tracing "can be thought of as the fraction of all contacts traced, assuming perfectly successful quarantine upon tracing, or the degree to which infectiousness of contacts is reduced assuming all of them are traced."
Hinch et al. 2020	Individual-based network model of an urban population of 1 million individuals	• 80% of symptomatic individuals self-quarantine together with all household members, irrespective of app ownership.<br>• Over 70s self-isolate ('shield') throughout.<br>• Incubation period 6 days (S.D. 2.5)<br>• Mean generation time 6 days<br>• Infectiousness varies over the time course of infection (gamma distribution, mean 6 days) • 18% of infections are asymptomatic<br>• 82% of infections are divided into severe and non-severe categories; differing proportions by age.<br>• Disease severity correlates with infectiousness; compared to mildly symptomatic individuals are taken to be 0.48 times as infectious as individuals with relatively severe symptoms, and asymptomatic individuals 0.29 times as infectious (Luo et al. 2020).<br>• Probabilities of hospitalisation, demand for critical care, rates of recovery and death (if infected) are all age dependent; 0.75% infection fatality ratio.<br>• Hospital interactions and transmission not modelled; hospitalised patients are removed from the	• Scenario 1: no app<br>• Scenario 2: app without recursion<br>○ Quarantine: index cases, their households, their contacts<br>○ Release: everybody after 14 days from notification<br>• Scenario 3: app with recursion<br>○ Quarantine: as scenario 2 plus household members of contacts<br>○ Release: as scenario 2<br>• Scenario 4: app with recursion and cluster release<br>○ Quarantine: as scenario 3<br>○ Release: as scenario 2&3 plus release of index case clusters if nobody from the cluster develops symptoms within 5 days<br>• Scenario 5: app with recursion and testing as follow-up<br>○ Quarantine: as scenario 3&4<br>○ Release: as scenario 2&3 plus release of an index case cluster if index case had a negative test

		interaction network and healthcare workers not incorporated.<br>• R_0 = 3.4 or 3 both modelled (corresponding to doubling times of 3 and 3.5 days)<br>• Base case: 80% app uptake amongst smartphone users (56% of the UK population), varied in sensitivity analysis.<br>• Smartphone ownership by 70% of the population<br>• The app has already collected a 7-day memory of contacts at the end of lockdown.<br>• 80% of modelled contacts with other app users are registered by the app.<br>• 100% initial compliance with quarantine on notification and drop-out rate of 2% per day.<br>• Symptomatic individuals quarantine for 7 days; asymptomatic household members and traced individuals quarantine for 14 days.<br>• Either 0.05%, 0.2% or 0.5% of app users declare symptoms for reasons unrelated to COVID-19 each day	• Scenario 6: app with recursion and notification upon testing<br>○ Quarantine: contacts are notified only after index case tests positive<br>○ Release: as scenario 2&3
Kim and Paul 2020	Derivation of mathematical equations applicable to automated contact tracing including a basis for determining minimum required app uptake to control spread.	• 75% of those who are infectious will be confirmed as such (base case, figure 2)<br>• Proximity radius r0 set at 2 metres<br>• P_t (probability of transmission of infection to any contact who is within the proximity radius r_0 – the specified minimum radius of contact - for longer than t_0, a specified minimum duration) treated as variable<br>• Assumes no environmental transmission<br>• Assumes limited transmission from asymptomatic or pre-symptomatic individuals (see detail for scenarios, right)<br>• Incubation period or the impact of temporal delays to isolation/quarantine not explicitly modelled	• Fraction of truly infected individuals confirmed as sick: 75-95%<br>• Fraction of infected individuals diagnosed as sick who will confirm their status via the app: 80 and 100%<br>• Minimum fraction of individuals at risk needing to be traced and quarantined to control pathogen spread: 60%,75% or 90%<br>• Fraction who enrol, transmission probability (P_t) and the fraction of individuals who test positive and confirm via the automated contact tracing system (f_c) are all varied from 0-100% (in graphs presented in figure 2)
Kucharski et al. 2020	Individual-level transmission model, stratified into four settings (household, work, school, other). Individuallevel contact distributions generated for 20,000 primary cases; secondary infections randomly generated with and without control measures in place.	• Secondary attack rate of 20% within household and 6% among other contacts, corresponding to an overall reproduction number of 2.6. (sensitivity analysed)<br>• Infectious period = 5 days (for cases that will become symptomatic, 1st day is pre-symptomatic) - implies a serial interval of ~6.5 days. (sensitivity analysed)<br>• Mean delay of 2.6 days from onset-to-isolation in baseline scenario, with a distribution from 0-4 days<br>• 40% of cases asymptomatic<br>• 50% relative infectiousness of asymptomatic cases<br>• 90% of symptomatic cases will eventually be tested and self-isolate<br>• 71% of UK population estimated to own a smartphone (based on 85% ownership amongst those aged 16+, with the 16% of the UK population <10 years old or over 80 years old)<br>• 75% app uptake amongst smartphone owners<br>• Individuals under age 10 or over 80 will not use a smartphone app<br>• Household quarantine alongside other measures in all contact tracing scenarios • 100% accurate contact detection amongst those with the app downloaded • 90% adherence to quarantine.<br>• Quarantine sufficiently fast to prevent any onwards transmission among successfully traced contacts, implying tracing and quarantine within around 2–3 days of exposure (based on He et al. 2020).<br>• Contact patterns simulated at random for each individual in our population (i.e. model does not incorporate correlation between an individuals' number of contacts and their infection risk)<br>• Assumed that routine self-isolation would not increase household transmission<br>• No clustering of contacts and that household contacts are the same people daily, but contacts outside home are made independently each day.<br>• In the self-isolation only scenario, assumed individuals no risk of onward transmission (even to household members) if successfully isolated. Otherwise assumed household quarantine was in place alongside other measures.<br>• Assumed that infected individuals would not test positive if tested >1 day pre-symptom onset.	• No control measures;<br>• Self-isolation of symptomatic cases away from their household;<br>• Self-isolation and household quarantine after onset of symptoms in primary case;<br>• Quarantine of work/school contacts;<br>• Manual tracing of acquaintances (i.e. contacts that have been met before); manual tracing of all contacts;<br>• App-based tracing;<br>• Mass testing of cases regardless of symptoms - assumed infected individuals would be identified and immediately self-isolate at a random point during or after 5-day infectious period<br>• A limit on daily contacts made in 'other' settings (with the baseline limit being 4 contacts), which is also applied in combination with both manual and app-based CONTACT TRACING scenarios; • A proportion of the adult population working from home.<br><br>Sensitivity analyses:<br>• No pre-symptomatic transmission<br>• Shorter delay to self-isolation<br>• Longer delay to self-isolation<br>• HH SAR=20%, other contact SAR=7%<br>• HH SAR=40%, other contact SAR=5%

Xia and Lee, 2020	Derivation of mathematical equations for upper and lower bounds on minimum adoption rate of a Bluetooth-based automated contact tracing app required for R_0<1	• The number of direct infections caused by a patient follows a Poisson distribution with mean R_0.<br>• Patients are independent and identically distributed with respect to showing severe enough symptoms to be tested.<br>• Newly identified cases (who are not detected as part of a cluster through contact tracing) are primarily identified due to clinical severity<br>• The proportion of all infected individuals detected in this way is between 2 and 10%<br>• All contacts will be detected and identified through the device (100% sensitivity).	• R_0 of 3,4,5 or 6<br>• Proportion of all infected individuals detected by the medical system (due to clinical severity) = 2, 5 or 10%
Yasaka et al. 2020	Transmission graph based random simulation model	• Maximum length of transmission paths = 3 (individuals that directly interacted with an infected individual would be assigned elevated risk levels, as well as their app-registered contacts and those contacts' app-registered contacts)<br>• Each individual is considered to be in one of the susceptible, infected, or recovered states; however, nodes are contact points, not individuals, with contact points arranged in layers to visualize spread across time, •<br>Individuals may move to a new contact point at each unit of time, forming a directed edge from the prior to the current contact point.<br>• Individuals may refrain from being at a contact point at any point in time, to model home isolation	• 0, 25, 50 and 75% app adoption.

Abbreviations and acronyms:

HH: household; R_0: basic reproduction number; $R_{symptomatic}$: basic reproduction number during the symptomatic stage of the illness; SAR: secondary attack rate; S.D. standard deviation

Table S2: Detailed lessons learned and other key findings

Reference	Lessons learnt from implementation of the intervention	Other key findings/conclusions
**1) Studies of fully-automated contact tracing**		
Bulchandani et al., 2020	N/A (modelling study)	For any given fraction of asymptomatic transmission, and any (non-zero) RS (a combined measure of the number of pre-symptomatic infections and the efficacy of quarantine) ≤ R0, there is a critical point of app uptake corresponding to the onset of "digital herd immunity". This is estimated at 75-95% (depending on the fraction of asymptomatic transmission).
Ferretti et al, 2020	N/A (modelling study)	The authors propose 8 requirements, in addition to the requirement that participation in the system should be voluntary, stating:<br>"Requirements for the intervention to be ethical and capable of commanding the trust of the public are likely to comprise the following:<br>i. Oversight by an inclusive and transparent advisory board, which includes members of the public.<br>ii. The agreement and publication of ethical principles by which the intervention will be guided. iii. Guarantees of equity of access and treatment. iv. The use of a transparent and auditable algorithm.<br>v. Integrating evaluation and research in the intervention to inform the effective management of future major outbreaks. vi. Careful oversight of and effective protections around the uses of data. vii. The sharing of knowledge with other countries, especially low- and middle-income countries. viii. Ensuring that the intervention involves the minimum imposition possible and that decisions in policy and practice are guided by three moral values: equal moral respect, fairness, and the importance of reducing suffering" (These three values are based on a 2018 report by the Nuffield Council on Bioethics).
Hinch et al., 2020	N/A (modelling study)	• In the UK, approximately 100,000 (PCR) tests per day would be required to permit 'smart release' from quarantine based on test results of the index case (with a doubling time of 3.5 days), or nearly 200,000 assuming a doubling time of 3 days or with quarantine being initiated based on a positive test result (and assuming a doubling time of 3.5 days)<br>• "In order to maintain low mortality, we recommend continued lockdown (shielding) of people aged over 70 - a group with assortative mixing, low smartphone use (approximately one quarter), and high COVID-19 mortality."
Kim and Paul 2020	N/A (modelling study)	• The authors conclude that 'in real-world scenarios, automated contact tracing alone cannot contain a pandemic driven by a pathogen like SARS-CoV-2'; this is primarily because of 'a large degree of spreading from pre-symptomatic and subclinical hosts, and the rapidity with which the virus spreads through proximity alone if no additional measures are taken to mitigate the spread'.<br>• The effectiveness of contact tracing via an app-based system (the ratio of the actual number of individuals notified to the minimum number that should be notified to control the spread of the disease) drops to 64% (80% x 80%) when the uptake rate is 80% of the threshold uptake required to bring R_0<1, and 25% (50% x 50%) when it is 50% of this threshold. This is because it affects both the proportion of sick individuals who can report their status via the system and the proportion of contacts who can be notified.<br>• All of these factors together with 'insufficient sampling due to limited participation amongst the population and possibly incomplete reporting of sick cases' limit automated contact tracing systems' efficacy.<br>• Reducing the transmission probability (per contact episode) and the increasing fraction of individuals that test positive can assist in reducing the burden on automated contact tracing.

Kucharski et al, 2020	N/A (modelling study)	• Estimate that at least 30–50 additional tests would be required for each case detected (meaning a large volume required daily if incidence high).<br>• Individual-level variation in transmission and contact networks are important considerations, as high variation can lead to superspreading events.<br>• Gatherings in other settings (e.g. mass gathering-type events) need to be restricted to relatively small sizes (fewer than 10-20 contacts) for a noticeable impact on transmission
Xia and Lee 2020	N/A (modelling study)	• To achieve disease control and enable a 'return to normalcy' the app adoption rate 'needs to be very high, e.g., above 95% depending on the disease parameters; with more vigilance in disease surveillance to detect mild cases earlier, the number may be brought down to about 90%.'
Yasaka et al. 2020	N/A (modelling study)	QR code approach requires user motivation - users may become fatigued from such behaviour over time and choose to discontinue or may be dissuaded from participating at the onset.<br>• Proposes the use of an individual-specific QR code to verify that a diagnosis is confirmed; these can only be generated by 'authorised users'. Mechanism for sharing these not detailed.<br>"Should a malicious user gain access to the system, this user would be able to do nothing more than manipulate the app by reporting false confirmed diagnoses."
**2) Studies of partially-automated contact tracing**		
Danquah et al, 2019	"Suggested improvements … included the need for better network coverage; … improved battery life and quality of phones; the need for further training on synchronising the data... between the phone and the server; the need for increased compensation to offset phone charging; better strategies for overcoming distances to charging booths; and more refresher training for contact tracing and monitoring using the study phones."	None of note
Li et al, 2017	"ICMS (infection control and management system) implementation took significant time and effort from users."	• Where 'some specific data analytics and workflow processes were not available' within the infection control management software, this 'necessitated workflow changes or workarounds'<br>•Some additional epidemiologically important organisms (e.g. Influenza A virus, respiratory syncytial virus) were tracked for the first time.
Schafer et al, 2016	• "Many avoidable problems were encountered in locations without IT staff or adequate IT knowledge, including improper set up and maintenance of the database and network, recurring network connectivity outages, poor data security, and malware epidemics."<br>• "Inconsistent electricity and unreliable Internet access made data entry and transmission challenging."<br>• "Problems related to insufficient infrastructure and IT glitches were often incorrectly attributed to … the Epi Info VHF application itself"<br>• "Particularly challenging was the attempt to accommodate the needs of multiple countries with 1 software product."	• The EpiInfo VHF app's contact tracing features 'were only used to their full extent in a few locations'.<br>• 'There was often reluctance to change from pre-existing paper-based systems.'<br>• Technical issues and problems related to insufficient infrastructure were often (incorrectly) attributed to the Epi Info VHF (Viral Haemorrhagic Fever) application itself<br><br>The authors also highlight that, compared to comparator systems: prevents contacts who have gone missing and could potentially be sick from being overlooked because their follow-up period is over; enables tracking of how many contacts were converted to cases and the proportion of cases who were previously identified as contacts for evaluation purposes.<br>It also collected data used to produce daily outbreak reports and to predict the course of future outbreaks.

Sacks et al, 2015	"careful consideration should be given to whether it is feasible and beneficial to implement new technology during an ongoing outbreak. When [such a] decision is made, it is critical to accompany the deployment with close managerial oversight to quickly correct data inconsistencies and to address challenges."<br>• "Messy data from the field that required additional editing in the backend of<br>CommCare was not easily accommodated in Tableau"<br>• Need for hardware and phone configuration support - "During the expansion phase, recruitment of local tech-savvy youth volunteers helped to accelerate the configuration of hundreds of phones."<br>• "time limitations were… a challenge as the dashboards were built by Tableau experts who were volunteer consultants. Commissioning full-time Tableau consultants could have helped to shorten the time required to build the dashboards; however, they may not have had as much experience as the volunteer experts suggested by Tableau Foundation.<br>• "While government staff members were especially enthusiastic about the wealth of data that could be used for supervision of contact tracers, actual use of the data was limited."<br>"Clearer standards [for contact tracing protocols and metrics] from the outset could have accelerated the design process.	• 86% agreement in second round of validation against reference database derived from paper-based forms (78% in the first round).<br>Additional benefits included:<br>• Pre-set contact tracing algorithms with built-in skip logic can guide a contact tracer through visits by prompting key questions based on previously inputted answers.<br>• Real-time data on performance (number of visits per day, GPS points, etc.) can inform supervisors for monitoring and supporting the contact tracers.<br>• Multimedia files including image, audio, and video can assist contact tracers in sensitization activities and can be used for refresher training as needed.<br>10
Tom-Aba et al, 2015	• "The deployment of the technology was not without some challenges and these<br>were the initial costs of setting up the required technology (getting the phones and tablets, preloading them with the required forms), the need for trained personnel (most of our contact tracers were residents of the Nigeria Field Epidemiology and Laboratory Training Programme and other public health professionals) many had participated in polio surveys, some had used the ODK before and most knew how to use smartphones and tablets) and the need to have highly effective internet connections to run the applications."<br>• "The integration of Epi-info VHF with form hub technology to align same data structure across the system posed some challenges."	Improvement in contact follow-up rates (from a range between 90-99% to consistently 100%) after introduction); however concurrent factors may also explain this
**3) Studies of automated contact detection in a relevant disease context (without subsequent contact tracing or contact notification)**		
Aiello et al, 2016	• Required "extensive mapping of on-campus routers, occasional debugging, data cleaning and verification."<br>• 95.2% participated due to the cash incentive	None of note
Al Qathrady et al, 2016	• The tracing system can identify all infected cases even if it starts the tracing process only knowing one case.<br>• Prioritisation of contacts for testing is more accurate (up to ~80% accuracy) when adopting either of two selection strategies; encounter infection probability, based on cumulative probability of infection based on previous recorded encounters with infected individuals, or a 'mostly named' strategy (Armbruster et al. 2007)[47] based on prioritising contacts identified as such by the largest number of index cases	The tracing system can identify all infected cases even if it starts the tracing process only knowing one case. Prioritisation of contacts for testing is more accurate (up to ~80% accuracy) when adopting either of two selection strategies; encounter infection probability, based on cumulative probability of infection based on previous recorded encounters with infected individuals, or a 'mostly named' strategy (Armbruster et al. 2007)[47] based on prioritising contacts identified as such by the largest number of index cases.
Voirin et al, 2015	"Most contacts (in this hospital-based study) occurred between nurses or between a nurse and a patient"	"Even with detailed contact and virological data, understanding whether or not transmission actually occurred remains a challenge because various other factors modulate the probability of transmission for both source and susceptible host. These factors include individual characteristics (eg, severity of disease, immunosuppression, immunosuppressive therapies, or influenza vaccination), microbial agent features (eg, virulence or inoculum size) and environment (eg, ward specialty or compliance with hygiene protocols), which could provide useful contextual information"

Abbreviations and acronyms:

IC: infection control; IT: information technology; N/A: not applicable; ODK: Open DataKit; QR: Quick Response code; UK: United Kingdom; PCR: polymerase chain reaction; VHF: Viral Haemorrhagic Fever

Table S3 – Effective Public Health Practice Tool Quality Assessment (Thomas et al., 2008; https://merst.ca/ephpp/)

	SELECTION BIAS			STUDY DESIGN			CONFOUNDERS			BLINDING			DATA COLLECTION METHODS			WITHDRAWALS AND DROP-OUTS			INTERVENTION INTEGRITY			ANALYSES				GLOBAL RATING FOR THIS PAPER
**Reference**	**Q1**	**Q2**	**Rate this section**	**Indicate the study design**	**. Q2**	**Rate this section**	**Q1**	**Q2**	**Rate this section**	**Q1**	**Q2**	**Rate this section**	**Q1**	**Q2**	**Rate this section**	**Q1**	**Q2**	**Rate this section**	**Q1**	**Q2**	**Q3**	**Q1**	**Q2**	**Q3**	**Q4**	
Aiello (iEpi sub-study only)	Somewhat likely	<60%	Weak	Sub-study (descriptive observational cohort) within a cluster-randomised controlled trial	No	N/A	N/A	N/A	N/A	N/A		N/A	Can't tell	Can't tell	Weak	Can't tell	80-100%	Moderate	N/A	N/A	No	N/A	Individual	Yes	N/A	Moderate
Danquah et al. 2019	Very likely	80-100%	Strong	Proof-of-concept study with phased introduction	No	N/A	No	N/A	Moderate	N/A	Can't tell	Weak	Can't tell	Can't tell	Weak	No	Can't tell	Weak	Can't tell	Can't tell	No	Individual	Individual	Yes	Can't tell	Weak
Voirin et al. 2015	Somewhat likely	80-100%	Mod-erate	Proof-of-concept observational cohort study	No	N/A	N/A	N/A	N/A	N/A	Can't tell	N/A	Can't tell	Can't tell	Weak	Yes	80-100%	Strong	N/A	N/A	N/A	N/A	Individual	Yes	N/A	Moderate

Full EPHPP question list:

SELECTION BIAS

(Q1) Are the individuals selected to participate in the study likely to be representative of the target population?

(Q2) What percentage of selected individuals agreed to participate?

Rate this section*

STUDY DESIGN

(Q1) Indicate the study design

(Q2) Was the study described as randomized? If NO, go to Component C.

(Q3) If Yes, was the method of randomization described? (See dictionary) – *excluded as not applicable in any included study*

(Q4) If Yes, was the method appropriate? (See dictionary) – *excluded as not applicable in any included study*

Rate this section*

CONFOUNDERS

(Q1) Were there important differences between groups prior to the intervention?

(Q2) If yes, indicate the percentage of relevant confounders that were controlled (either in the design (e.g. stratification, matching) or analysis)?

Rate this section*

BLINDING

(Q1) Was (were) the outcome assessor(s) aware of the intervention or exposure status of participants?

Q2) Were the study participants aware of the research question?

Rate this section*

DATA COLLECTION METHODS

(Q1) Were data collection tools shown to be valid?

(Q2) Were data collection tools shown to be reliable?

Rate this section*

WITHDRAWALS AND DROP-OUTS

(Q1) Were withdrawals and drop-outs reported in terms of numbers and/or reasons per group?

(Q2) Indicate the percentage of participants completing the study. (If the percentage differs by groups, record the lowest).

Rate this section

INTERVENTION INTEGRITY

(Q1) What percentage of participants received the allocated intervention or exposure of interest?

(Q2) Was the consistency of the intervention measured?

(Q3) Is it likely that subjects received an unintended intervention (contamination or co-intervention) that may influence the results?

ANALYSES

(Q1) Indicate the unit of allocation (select one)

(Q2) Indicate the unit of analysis (select one)

(Q3) Are the statistical methods appropriate for the study design?

(Q4) Is the analysis performed by intervention allocation status (i.e. intention to treat) rather than the actual intervention received?

---

* Guidance to support the assignment of ratings for each section and the global study rating is available at https://www.ephpp.ca/PDF/QADictionary_dec2009.pdf

Table S4 – Quality assessment using adapted CHEERS Checklist (Husereau et al. 2013) (modelling studies only)

	Question number											
**Reference**	**2**	**3**	**4**	**5**	**7**	**15**	**16**	**17**	**18**	**22**	**23**	**24.**
Al Qathrady et al. 2016	Yes	Yes	No	Limited	N/A	Limited; figure provided	Yes (limited; parameters selected appear arbitrary and are not referenced)	Yes, although limited data analysed; observed contact network data is used directly in a simulation model of disease propagation	Limited (e.g. no references to justify parameter values)	Partial (no discussion of relationship of the findings to current scientific knowledge)	No	No
Bulchandani et al. 2020	Yes	Yes	No	No	Yes (different levels of uptake)	Yes (figure also provided)	Yes (however some assumptions not justified/referenced)	Limited	Yes; limited (parameter uncertainty not incorporated)	Yes	No	No
Ferretti et al. 2020	Yes	Yes	No	Limited	Yes	Yes	Yes (in reference 9)	Yes; however methods for incorporating population heterogeneity not detailed	Yes	Yes	Yes	Yes
Hinch et al. 2020	No	Yes	Yes	Yes (combination of 'small world' and random networks)	Yes	Yes	Yes	Yes	Yes (see parameter sheet[1])	Yes	No	No
Kim and Paul 2020	Yes	Yes	No	Limited	N/A	Limited	Yes (limited)	Limited; methods for incorporating population heterogeneity not detailed, uncertainty not accounted for	Limited	Partial (no discussion of relationship of the findings to current knowledge)	Yes	No
Kucharski et al. 2020	Yes	Yes	Yes	Yes	Yes	Yes (figure also provided)	Yes	Yes	Yes; limited (parameter uncertainty not incorporated)	Yes	Yes	Yes
Xia and Lee 2020	Yes	Yes	No	No	N/A	No	Yes (limited; rationale for these not always explained)	Limited; methods for incorporating population heterogeneity and contact networks not detailed	Yes; limited (parameter uncertainty incorporated only for two input variables)	Yes	No	No
Yasaka et al. 2020	Yes	Yes	No	No	Yes (different levels of uptake)	No	Yes (limited)	No	No	Yes	No	Yes

N.B. Responses to questions 1, 6, 8-14 and 19-21 (indicated in italics below) are not presented as they were not applicable in all studies assessed. Responses to question 1 are also not presented as none of these studies were economic evaluation studies. Other terms in italics were considered only if applicable to the study in question.

---

[1] Table: Parameter dictionary https://github.com/BDI-pathogens/OpenABM-Covid19/blob/master/documentation/parameters/parameter_dictionary.md

Question descriptors:

1 Identify the study as an economic evaluation or use more specific terms such as "cost-effectiveness analysis", and describe the interventions compared.

2 Abstract: Is a structured summary of objectives, perspective, setting, methods (including study design and inputs), results (including base case and uncertainty analyses), and conclusions provided?

3 Is there an introduction which provides an explicit statement of the broader context for the study and presents the study question and its relevance for health policy or practice decisions?

4 Target population and subgroups: Are the characteristics of the base case population and subgroups analysed described, including why they were chosen?

5 Setting and location: are relevant aspects of the system(s) in which the decision(s) need(s) to be made stated?

6 Study perspective: are the perspective of the study described and related to the costs being evaluated?

7 Comparators: are the interventions or strategies being compared described and why they were chosen stated?

8 Are the time horizon(s) over which costs and consequences are being evaluated and why appropriate stated?

9 Is the choice of discount rate(s) used for costs and outcomes and why appropriate reported?

10 Are the health outcomes used as the measure(s) of benefit in the evaluation and their relevance for the type of analysis performed described?

11a Single study-based estimates: Are the design features of the single effectiveness study and why the single study was a sufficient source of clinical effectiveness data fully described?

11b Synthesis-based estimates: Are the methods used for identification of included studies and synthesis of clinical effectiveness data fully described?

12 If applicable, are the population and methods used to elicit preferences for outcomes described?

13a Single study-based economic evaluation: are approaches used to estimate resource use associated with the alternative interventions described? Are primary or secondary research methods for valuing each resource item in terms of its unit cost described? Are any adjustments made to approximate to opportunity costs described?

13b Model-based economic evaluation: are approaches and data sources used to estimate resource use associated with model health states described? Are primary or secondary research methods for valuing each resource item in terms of its unit cost described? Are any adjustments made to approximate to opportunity costs described?

14 Currency, price date and conversion: Are the dates of the estimated resource quantities and unit costs reported? Are methods for adjusting estimated unit costs to the year of reported costs described, if necessary? Are methods for converting costs into a common currency base and the exchange rate described?

15 Choice of model - do the authors describe and give reasons for the specific type of decision analytical model used? (Providing a figure to show model structure is strongly recommended).

16 Do the authors describe all structural or other assumptions underpinning the decision-analytical model?

17 Are all analytical methods supporting the evaluation (/model) described? This could include methods for dealing with skewed, missing, or censored data; extrapolation methods; methods for pooling data; approaches to validate or make adjustments (such as half cycle corrections) to a model; and methods for handling population heterogeneity and uncertainty.

18 Do the authors report the values, ranges, references, and, if used, probability distributions for all parameters? Do the authors report reasons or sources for distributions used to represent uncertainty where appropriate? (Providing a table to show the input values is strongly recommended).

19. For each intervention, are mean values for the main categories of estimated costs and outcomes of interest, as well as mean differences between the comparator groups, reported? If applicable, are incremental cost-effectiveness ratios reported?

20a Single study-based economic evaluation: Do the authors describe the effects of sampling uncertainty for the estimated incremental cost and incremental effectiveness parameters, together with the impact of methodological assumptions (such as discount rate, study perspective)?

20b Model-based economic evaluation: Do the authors describe the effects on the results of uncertainty for all input parameters, and uncertainty related to the structure of the model and assumptions?

21 If applicable, do the authors report differences in costs, outcomes, or cost-effectiveness that can be explained by variations between subgroups of patients with different baseline characteristics or other observed variability in effects that are not reducible by more information?

22 Do the authors summarise key study findings and describe how they support the conclusions reached? Do the authors discuss limitations and the generalisability of the findings and how the findings fit with current knowledge?

23 Do the authors describe how the study was funded and the role of the funder in the identification, design, conduct, and reporting of the analysis and describe other nonmonetary sources of support?

24 Do the authors describe any potential for conflict of interest of study contributors in accordance with journal policy. In the absence of a journal policy, we recommend authors comply with International Committee of Medical Journal Editors recommendations.

Table S5 Quality assessment notes – case studies only

Reference	Selection bias	Information bias	Confounding risk	Selective reporting	Funding detailed	Conflicts of interest reported? Any which may have influenced the study?
Danquah et al. 2019	**Likely**; reasons for use of appbased system (vs. paper-based system only) for any given case not clear	**Likely** (data highly incomplete, particularly for paper-based system; reasons for this unclear)	**Possible** (reasons for use of app-based vs. paper-based system only unclear; risk difficult to assess)	Can't tell; appears unlikely (detailed methods and data presented)	Yes	Reported; none
Li et al. 2017	**Unlikely** (applied across whole hospital)	**Unlikely** (summary estimates from routine data)	**Possible** (before-and-after comparison; other factors may have changed between the two time periods)	Can't tell; limited detail available due to nature of paper (abstract and conference poster)	No	Not reported
Schafer et al. 2016	**Likely**; uptake variable and reasons not detailed (however no comparative analyses therefore impact on this on findings limited)	**Difficult to assess**; few quantitative outcomes reported	**Not applicable** (no comparative analyses)	Can't tell; appears unlikely	Yes	Reported; none
Sacks et al. 2015	**Likely**; reasons for and process of selecting the 5 prefectures involved not clear, participation by individual contact tracers may introduce further selection bias (limited detail available)	**Unlikely** (routine data from app); however agreement with paper-based database was 78% then 86% in successive rounds of validation	**Not applicable** (no comparative analyses)	Can't tell	Yes (for the contact tracing program; research funding not detailed)	Reported; none
Tom-Aba et al. 2015	**Unlikely** (article suggest implemented across whole contact tracing workforce in Nigeria)	**Unlikely** (routine data from app)	**Possible** (before-and-after comparison; other factors may have changed between the two time periods, which the authors acknowledge; risk difficult to assess)	Can't tell	Yes	Reported; none

Table S6: Studies selected for level 2 (full-text) screening which have no full-text available in English:

Authors, publication details, DOI	Title	Language of full-text manuscript	Abstract
Amela-Heras C., García M.C. and Sierra Moros M. J., Revista Espanola de Salud Publica. 2010;84(5):497-506. DOI: 10.1590/s113557272010000500004	Monitoring and non Pharmacologic Measures during a Pandemic virus (H1N1) 2009 in Spain / Bases epidemiológicas para la toma de decisiones sobre medidas de salud pública (no farmacológicas) durante la respuesta a la pandemia de gripe (H1N1) 2009	Spanish	Nonpharmacological public health measures are used to reduce exposure of susceptible persons to an infectious agent. Its use is recommended at the start of a pandemic, when the transmission begins, and the characteristics of the new virus are unknown. The National Plan for Preparedness and Response to Pandemic Influenza included the application of these measures, recommending the establishment of an Advisory Committee for implementation, with a multidisciplinary composition. The mandate at this Committee is to analyze the epidemiological and social context in confronting the pandemic and to propose public health measures according to their evolution.<br>This article describes isolation, quarantine and closure of schools measures, aiming to reduce the spread of the virus in the population.<br>It also reviews the epidemiological parameters that help to understand the impact of its implementation.<br>The public health measures reviewed in this paper reduce transmission of the virus, and they have to be considered in response to an influenza pandemic. The impact on health will depend on how quickly they are taken and how people accept and follow them. Response plans should recommend its use, depending on the severity and characteristics of the new pandemic virus.<br>The data analysis should be considered as part of the response, because the information collection and analysis will be key to advising health authorities on what measures should be adopted.
Ren H., Yuan Z.A., Gu Z.R., Hu J.Y., Wang Y., Li Y.T. Zhonghua Yu Fang Yi Xue Za Zhi. 2013 Jan;47(1):63-6. DOI: not available	Study and application of transmission tracking analysis technique during incubation period of respiratory infectious diseases.	Chinese	OBJECTIVE:<br>To develop a new transmission tracking analysis technique during incubation period of respiratory infectious diseases, and to discuss its practical value in the field survey of infectious diseases.<br>METHODS:<br>The classical epidemiological theory was integrated with geographic information system. The transmission tracking analysis technique was established based on the modelling platform ArcGIS Engine Developer Kit 9.3, using the techniques of address matching, shortest path analysis and buffer analysis, and programming by Visual C++. Eight serious sever acute respiratory syndrome (SARS) cases in Shanghai in year 2003 were then chose as prototype to set up the test cases A-H. The electronic map and population density data were separately collected from Institute of Surveying and Mapping in Shanghai and Shanghai statistical yearbook 2003, to calculate and explore the parameters as length of transmission path, area of buffer zone and key departments by single and multi case analysis module.<br>RESULTS:<br>The single case transmission tracking analysis showed that the length of transmission track of case A was 129.89 km during April 25th to 29th in 2003, including 12 tracing point and 108 intimate contacts, and the total area of buffer zone was 7.11 km(2) including 81 important institutes, naming 72 schools, 6 kindergartens and 3 gerocomiums. The multi-case transmission tracking analysis showed that the 8 cases shared 5 tracks without any temporal communication. However, there was a spatial communication whose length was 1.42 km and area was 0.60 km(2). There were no important institutes found in this communication area.<br>CONCLUSION:<br>Transmission tracking technique is practicable and efficient to trace the source of infection, analyze the transmission tracks, establish the isolation buffer area and explore the important geographic positions in epidemiological investigation.

References

Armbruster, Benjamin, and Margaret L. Brandeau. ”Who do you know? A simulation study of infectious disease control through contact tracing.” Proceedings of the 2007 Western Multiconference on Computer Simulation. 2007

Aiello AE, Simanek AM, Eisenberg MC, et al. Design and methods of a social network isolation study for reducing respiratory infection transmission: The eX-FLU cluster randomized trial. Epidemics 2016; 15: 38–55.

Al Qathrady M, Helmy A, Almuzaini K. Infection tracing in smart hospitals. IEEE, 2016: 1–8.

Amela-Heras C., García M.C. and Sierra Moros M. J., Revista Espanola de Salud Publica. 2010;84(5):497-506. DOI: 10.1590/s1135-57272010000500004 Bulchandani

VB, Shivam S, Moudgalya S, Sondhi S. Digital herd immunity and COVID-19. ArXiv Prepr ArXiv200407237 2020.

Danquah LO, Hasham N, MacFarlane M, et al. Use of a mobile application for Ebola contact tracing and monitoring in northern Sierra Leone: a proof-of-concept study. BMC Infect Dis 2019; 19: 810–810.

Ferretti L, Wymant C, Kendall M, et al. Quantifying SARS-CoV-2 transmission suggests epidemic control with digital contact tracing. Science 2020; published online March. DOI:10.1126/science.abb6936.

He X, Lau EH, Wu P, Deng X, Wang J, Hao X, Lau YC, Wong JY, Guan Y, Tan X, Mo X. Temporal dynamics in viral shedding and transmissibility of COVID-19. Nature Medicine. 2020 Apr 15:1-4.

Hinch R, Probert W, Nurtay A, et al. Effective Configurations of a Digital Contact Tracing App: A report to NHSX. 2020 https://github.com/BDI-pathogens/covid19_instant_tracing (accessed April 15, 2020).

Husereau D, Drummond M, Petrou S, et al. Consolidated health economic evaluation reporting standards (CHEERS) statement. Int J Technol Assess Health Care 2013; 29: 117–22.Kim H, Paul A. Contact Tracing: a game of big numbers in the time of COVID-19. ArXiv Prepr ArXiv200410762 2020.

Kucharski AJ, Klepac P, Conlan A, et al. Effectiveness of isolation, testing, contact tracing and physical distancing on reducing transmission of SARS-CoV-2 in different settings. medRxiv 2020.

Li J., Meng Q.S., Liu M., Chua E., Tan T.Y. Infection control software: Placebo or panacea? Antimicrob Resist Infect Control 2017; 6. DOI:10.1186/s13756-017-0201-4.

Luo L, Liu D, Liao XL, Wu XB, Jing QL, Zheng JZ, Liu FH, Yang SG, Bi B, Li ZH, Liu JP. Modes of Contact and Risk of Transmission in COVID-19: A Prospective Cohort Study 4950 Close Contact Persons in Guangzhou of China.

Nuffield Council on Bioethics, Research in Global Health Emergencies: Ethical Issues (28 January 2020); www.nuffieldbioethics.org/publications/research-in-global-healthemergencies.

Ren H., Yuan Z.A., Gu Z.R., Hu J.Y., Wang Y., Li Y.T. Zhonghua Yu Fang Yi Xue Za Zhi. 2013 Jan;47(1):63-6.

Sacks JA, Zehe E, Redick C, et al. Introduction of Mobile Health Tools to Support Ebola Surveillance and Contact Tracing in Guinea. Glob Health Sci Pract 2015; 3: 646–59.

Schafer IJ, Knudsen E, McNamara LA, Agnihotri S, Rollin PE, Islam A. The Epi Info Viral Hemorrhagic Fever (VHF) Application: A Resource for Outbreak Data Management and Contact Tracing in the 2014-2016 West Africa Ebola Epidemic. J Infect Dis 2016; 214: S122–36.

Thomas B, Ciliska D, Dobbins M, Micucci S. Quality assessment tool for quantitative studies dictionary: the Effective Public Health Practice Project (EPHPP). McMaster Univ 2008. https://merst.ca/ephpp/ (accessed April 21, 2020).

Tom-Aba D, Olaleye A, Olayinka AT, et al. Innovative Technological Approach to Ebola Virus Disease Outbreak Response in Nigeria Using the Open Data Kit and Form Hub Technology. PloS One 2015; 10: e0131000–e0131000.

Voirin N, Payet C, Barrat A, et al. Combining high-resolution contact data with virological data to investigate influenza transmission in a tertiary care hospital. Infect Control Hosp Epidemiol 2015; 36: 254–60.

Xia Y, Lee G. How to Return to Normalcy: Fast and Comprehensive Contact Tracing of COVID-19 through Proximity Sensing Using Mobile Devices. ArXiv Prepr ArXiv200412576 2020.

Yasaka TM, Lehrich BM, Sahyouni R. Peer-to-Peer Contact Tracing: Development of a Privacy-Preserving Smartphone App. JMIR MHealth UHealth 2020; 8: e18936– e18936.
